# The role of 25-hydroxyvitamin-D3 and vitamin D receptor gene in human periodontal ligament fibroblasts as response to orthodontic compressive strain: an in vitro study

**DOI:** 10.1186/s12903-021-01740-8

**Published:** 2021-08-06

**Authors:** Erika Calvano Küchler, Agnes Schröder, Vinicius Broska Teodoro, Ute Nazet, Rafaela Scariot, Gerrit Spanier, Peter Proff, Christian Kirschneck

**Affiliations:** 1 Department of Orthodontics, University of Regensburg, Franz-Josef-Strauss-Allee 11, 93053 Regensburg, Germany; 2 Private Practice Curitiba, Paraná, Brazil; 3 Department of Stomatology, Federal University of Paraná, Curitiba, Paraná Brazil; 4 Department of Maxillofacial Surgery, University of Regensburg, Regensburg, Germany

**Keywords:** Vitamin D, Tooth movement, Polymorphism, Gene

## Abstract

**Background:**

This study aimed to investigate, if different physiological concentrations of vitamin D (25(OH)D_3_) and single nucleotide polymorphisms in vitamin D receptor (*VDR*) gene have an impact on gene expression in human periodontal ligament (hPDL) fibroblasts induced by simulated orthodontic compressive strain.

**Methods:**

A pool of hPDL fibroblasts was treated in absence or presence of 25(OH)D_3_ in 3 different concentrations (10, 40 and 60 ng/ml). In order to evaluate the role of single nucleotide polymorphisms in the *VDR* gene, hPDL fibroblasts from 9 patients were used and treated in absence or presence of 40 ng/ml 25(OH)D_3_. Each experiment was performed with and without simulated orthodontic compressive strain. Real-time PCR was used for gene expression and allelic discrimination analysis. Relative expression of dehydrocholesterol reductase (DHCR7), Sec23 homolog A, amidohydrolase domain containing 1 (AMDHD1), vitamin D 25-hydroxylase (CYP2R1), Hydroxyvitamin D-1-α hydroxylase, receptor activator of nuclear factor-κB ligand (RANKL), osteoprotegerin (OPG), cyclooxygenase-2 (COX-2) and interleukin-6 (IL6) was assessed. Three single nucleotide polymorphisms in *VDR* were genotyped. Parametric or non-parametric tests were used with an alpha of 5%.

**Results:**

*RANKL, RANKL:OPG* ratio, *COX-2, IL-6, DHCR7, CYP2R1* and *AMDHD1* were differentially expressed during simulated orthodontic compressive strain (*p* < 0.05). The *RANKL:OPG* ratio was downregulated by all concentrations (10 ng/ml, 40 ng/ml and 60 ng/ml) of 25(OH)D_3_ (mean = 0.96 ± 0.68, mean = 1.61 ± 0.66 and mean = 1.86 ± 0.78, respectively) in comparison to the control (mean 2.58 ± 1.16) (*p* < 0.05). *CYP2R1* gene expression was statistically modulated by the different 25(OH)D_3_ concentrations applied (*p* = 0.008). Samples from individuals carrying the GG genotype in rs739837 presented lower *VDR* mRNA expression and samples from individuals carrying the CC genotype in rs7975232 presented higher *VDR* mRNA expression (*p* < 0.05).

**Conclusions:**

Simulated orthodontic compressive strain and physiological concentrations of 25(OH)D_3_ seem to regulate the expression of orthodontic tooth movement and vitamin-D-related genes in periodontal ligament fibroblasts in the context of orthodontic compressive strain. Our study also suggests that single nucleotide polymorphisms in the *VDR* gene regulate VDR expression in periodontal ligament fibroblasts in the context of orthodontic compressive strain.

**Supplementary Information:**

The online version contains supplementary material available at 10.1186/s12903-021-01740-8.

## Introduction

The periodontal ligament (PDL) is a connective tissue located between the cementum of teeth and the alveolar bone and mainly composed of fibroblast-like cells, characterized by collagen production, but also possessing some osteoblastic features [1]. The PDL actively participates in alveolar bone remodeling, which is the key component of orthodontic tooth movement (OTM) to therapeutically correct the position of misaligned teeth within the alveolar bone of the upper and lower jaws [2]. OTM is induced by the application of a mechanical force to a tooth by orthodontic appliances leading to the formation of tensile and pressure zones within the PDL [3]. As a reaction, PDL fibroblasts produce several pro-inflammatory mediators, when stimulated mechanically [4–8], leading to a sterile inflammatory reaction within the PDL, which ultimately induces osteoclast differentiation and activity [3]. PDL fibroblasts thus play a major role in mediating the molecular processes required for OTM [9].

Previous studies in orthodontics evaluated many factors that could be accountable for individual variations in the tissue response to OTM therapy [10–12]. Among these, vitamin D is known to regulate osteogenic differentiation in the PDL [13], affecting the adjacent alveolar bone [14], and previous studies reported a vitamin-D-associated enhancement of OTM [10–12, 15] as well as a reduced tendency for relapse after OTM [15]. Vitamin D is a liposoluble secosteroid essential for the body’s bone balance [16]. To become metabolically active, vitamin D is first converted to 25(OH)D_3_, also known as calcifediol, which is then converted into the active form of vitamin D calcitriol (1,25(OH) 2D_3_) [17]. The biological effects of vitamin D are mediated by binding to its intracellular receptor, the vitamin D receptor (VDR), a member of the nuclear receptor superfamily [18]. Additionally to the *VDR*, some other vitamin D-related genes are also closely related to the synthesis, activation and degradation of vitamin D such as *7-dehydrocholesterol reductase *(*DHCR7*),* Sec23 homolog A *(*SEC23A*),* amidohydrolase domain containing 1 *(*AMDHD1*),* vitamin D 25-hydroxylase *(*CYP2R1*) and *Hydroxyvitamin D-1-α hydroxylase *(*CYP27B1*) [17, 19]. VDR mediates the activities of vitamin D binding sites in the DNA stimulating the physiological regulation of several genes [17, 18], including *receptor activator of nuclear factor-κB ligand *(*RANKL*)*, osteoprotegerin *(*OPG*)*, cyclooxygenase-2 *(*COX-2*)* and interleukin-6 *(*IL6*)*,* which are involved in OTM [8, 20, 21].

The gene encoding the VDR in humans is located on chromosome 12q13.11, which spans ~ 100 kb and has five promotors, eight coding exons and six untranslated exons [22]. The *VDR* gene is known to exhibit many polymorphic regions [23, 24], including single nucleotide polymorphisms (SNPs), which influence the expression/functions of *VDR* and have been associated with complex traits, including oral phenotypes, such as periodontal disease [25, 26] and external apical root resorption as a sequela of orthodontic treatment [27].

Vitamin D presence [28, 29] and *VDR* expression [13, 14, 30–32] were observed in human periodontal soft tissues and cells. Furthermore, animal model studies indicate that vitamin D deficiency as well as its therapeutic supplementation or local administration can impact on the rate of OTM and the stability of tooth position after orthodontic treatment [33]. As of now, however, it is not clear, how vitamin D impacts gene expression pattern of PDL fibroblasts in the context of simulated orthodontic compressive strain and whether SNPs in the *VDR* genes can account for individual expression differences. Our hypothesis is that vitamin D levels and SNPs in the *VDR* gene influence gene expression during OTM. Therefore, the purpose of the present study was to investigate, if different physiological concentrations of 25(OH)D_3_ influence the expression of OTM-related genes and vitamin-D-related genes in human PDL (hPDL) fibroblasts as response to simulated orthodontic compressive strain and whether common SNPs in *VDR* are involved in individual variations of gene expression pattern.

## Methods

This in vitro study aimed to investigate the influence of physiological concentrations of 25(OH)D_3_ and SNPs in *VDR* in simulated orthodontic compressive strain. The ethics committee of the University of Regensburg, Germany, approved the collection of the samples and the subsequent experiments (Approval No. 12-170-0150). Informed written consent was obtained with an assent document from all subjects.

### In vitro setup for cell culture experiments

**Fig. 1:**
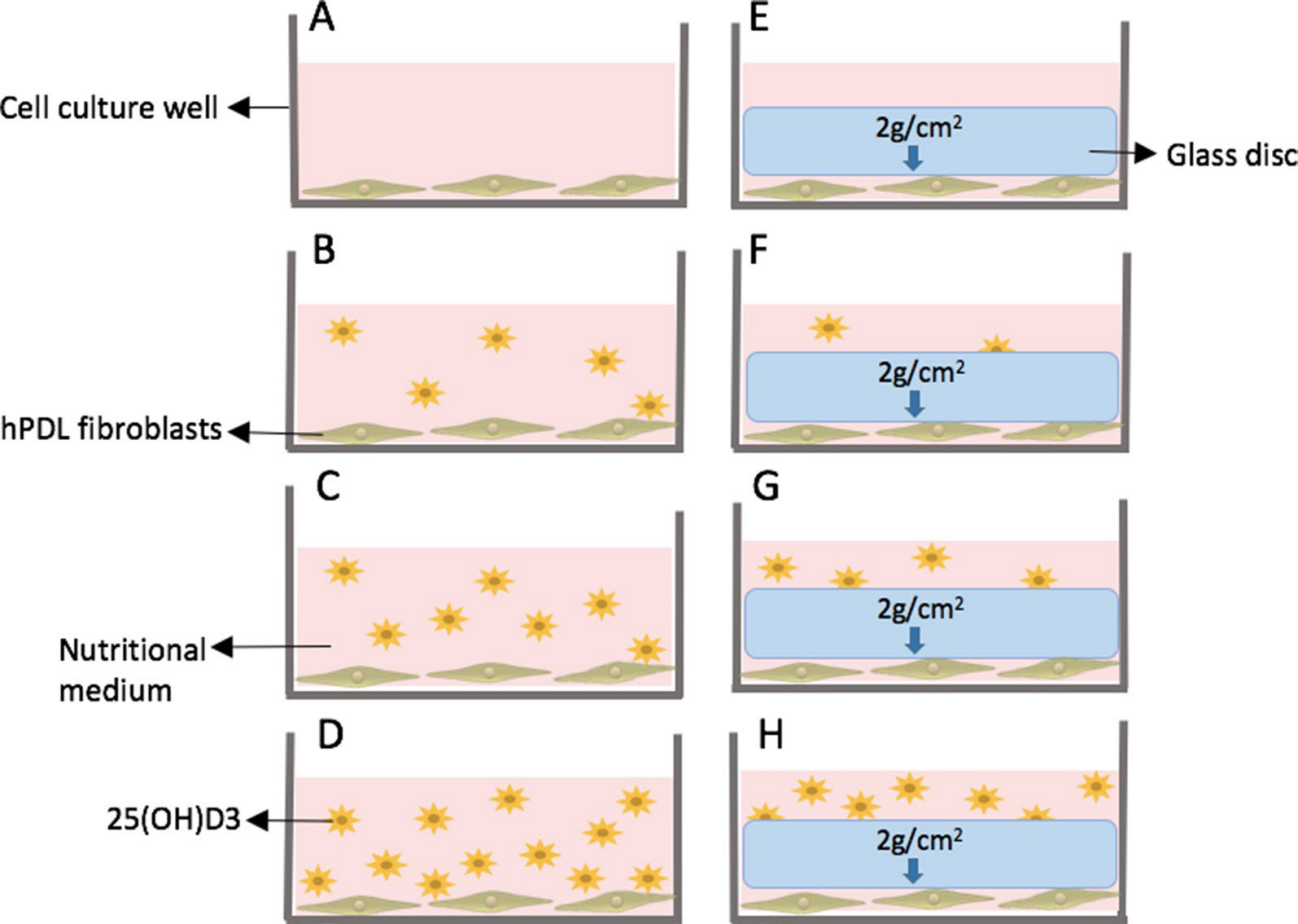
Experimental design. **A** Untreated physiological control. **A**–**D** No pressure groups (left side). **E**–**H** Simulated orthodontic compressive strain of 2 g/cm^2^ applied by a 17.1 g glass disc (right side). **A**, **E** No 25(OH)D_3_. **B**, **F** Treated with 10 ng/ml of 25(OH)D_3_. **C**, **G** Treated with 40 ng/ml of 25(OH)D_3_. **D**, **H** Treated with 60 ng/ml of 25(OH)D_3_

For this experiment, hPDL fibroblasts from periodontal connective tissue were used. hPDL from permanent caries-free teeth, extracted in a routine dental treatment at the maxillofacial surgery clinic at the University of Regensburg, were collected, isolated, cultivated and characterised according to an established method and protocol previously published [5, 6]. Briefly, hPDL fibroblasts from the third to fifth passages were seeded at a density of 70.000 cells per well into standard six-well cell culture plates. To simulate orthodontic compressive strain in hPDL pressure areas, a physiological compressive force of 2 g/cm^2^ was applied to the hPDL fibroblasts under cell culture conditions at 70% confluency for 48 h, using a glass disc [5, 6]. Figure 1 shows the setup of the in vitro experiment. Two experimental designs were carried out:

1. Evaluation of the effects of different concentrations of 25(OH)D_3_ during orthodontic compressive strain—stimulation of hPDL fibroblasts pooled from six patients, as previously established and described [6, 8], was performed either in absence or presence of 25(OH)D_3_ (1 mg, Tocris/Bio-Techne, Wiesbaden, Germany) in 3 different concentrations established according to The Endocrine Society's Clinical Guidelines [34]: 10 ng/ml (vitamin-D-deficient), 40 ng/ml (vitamin-D-sufficient) and 60 ng/ml (vitamin-D-sufficient simulating supplementation) (Fig. 1). Each of the experimental groups included three samples and three wells, with and without simulated orthodontic compressive strain. After 48 h mRNA expression levels of OTM-related genes and vitamin-D-related genes were evaluated.
2. Influence of SNPs in *VDR* gene on the cellular response to orthodontic compressive strain—stimulation was performed in individual samples from nine patients/teeth in triplicates (three wells) either in absence or presence of 40 ng/ml of 25(OH)D_3_ (1 mg, Tocris/Bio-Techne, Wiesbaden, Germany). Each experimental group included three wells, with and without simulated orthodontic compressive strain. After 48 h mRNA expression levels of *VDR* and the SNPs FokI, BglI and Apal in *VDR* were evaluated.

### Total RNA isolation and quantification of relative gene expression (RT-qPCR)

Total RNA from hPDL fibroblasts was extracted using 1 ml peqGOLD TriFast™ (PEQLAB Biotechnology GmbH, Erlangen, Germany) according to the manufacturer’s instructions. The RNA was instantaneously cooled on ice. For quantification and purity evaluation of the total RNA, optical density was photometrically measured at 280 nm, 260 nm and 230 nm (NanoPhotometer N60, Implen, Munich, Germany), as previously described [5, 6].

For complementary DNA (cDNA) synthesis, a standardized amount of 500 ng RNA per sample was transcribed and RT-qPCR amplification was performed with a Mastercycler® ep realplex-S thermocycler (Eppendorf AG, Hamburg, Germany) [5, 6]. Quantification cycle (Cq) values were determined as second derivative maximum of the fluorescence signal curve as previously described [35] using the software Realplex (version 2.2, Eppendorf AG, CalqPlex algorithm, Automatic Baseline, Drift Correction On) and the arithmetic mean of each duplicate Cq per gene and sample was used. For normalization of target genes, PPL22 and PPIB previously established reference genes stable in hPDL fibroblasts were used [5]. The studied target genes and reference genes are described in the Additional file 1: Table S1.

Relative gene expression was calculated as 2^−ΔCq^ with ΔCq = Cq (target gene) – Cq (mean RPL22/PPIB), divided by the respective arithmetic 2^−ΔCq^ mean of the untreated controls (for each experimental group) to established the relative gene expression. The gene-specific primers were designed according to MIQE quality guidelines using NCBI Primer-BLAST and additional software. The primers were synthesized and purified by Eurofins MWG Operon LLC (Huntsville, AL, USA; High Purity Salt Free Purification HPSF®). For each primer pair and qPCR run a no template control without cDNA was tested to assess a possible bias in results by primer dimers or contaminating DNA.

### Genomic DNA isolation and allelic discrimination analysis

Genomic DNA (gDNA) of hPDL cells of 9 patients was extracted using the GenElute Mammalian Genomic DNA Miniprep kit (Sigma Aldrich, Munich, Germany) according to the manufacturer's instructions. For purity evaluation and DNA quantification optical density was photometrically measured at 260 nm and 230 nm (NanoPhotometer N60, Implen, Munich, Germany). The OD_260nm/280 nm_ ratio > 1.8 indicated protein-free DNA.

The SNPs rs2228570 (FokI, A > G/Met > Thr), rs739837 (BglI, G > T/intronic) and rs7975232 (ApaI, A > C/intronic) in *VDR* were selected based on their minor allele frequency and their previously reported association. Genotyping was performed by allelic discrimination real-time PCR using the TaqMan assay in the Mastercycler® ep realplex-S thermocycler (Eppendorf AG, Hamburg, Germany) as described before [8].

### Statistical analysis

The software GraphPad Prism 8.0.1 (GraphPad Software Inc., San Diego, USA) was used for statistical analyses. Prior to the statistical analysis, all absolute data values were divided by the respective arithmetic mean of the respective untreated control group to obtain normalised data values relative to the values of the controls, which were set to 1. The Shapiro–Wilk test was used to assess the normality of the gene expression data and Levene's test to determine homogeneity of variance across groups. Parametric tests (ANOVA and t tests) were used to compare relative gene expression between groups. Non-parametric tests (Kruskal–Wallis in the co-dominant model and Mann–Whitney in the recessive model) were used to compare relative gene expression between individual sample genotypes. Post-hoc tests were performed using Tukey’s (ANOVA) or Dunn's (Kruskal–Wallis) tests. Statistical significance was established at *p* < 0.05.

## Results

### Effects of simulated orthodontic compressive strain and 25(OH)D_3_ on the expression of OTM-related genes and vitamin-D-related genes

Simulated orthodontic compressive strain impacted on gene expression. A statistically significant overexpression during simulated orthodontic compressive was observed for RANKL (*p* = 0.002 at 0 ng/ml and *p* = 0.001 at 60 ng/ml); the RANKL:OPG ratio (*p* = 0.01 at 0 ng/ml, *p* = 0.042 at 40 ng/ml and *p* = 0.033 at 60 ng/ml); COX-2 (*p* = 0.002 at 0 ng/ml, *p* = 0.001 at 10 ng/ml, *p* = 0.002 at 40 ng/ml and *p* = 0.019 at 60 ng/ml); IL-6 (*p* = 0.002 at 0 ng/ml, *p* = 0.002 at 10 ng/ml, *p* = 0.028 at 40 ng/ml and *p* = 0.038 at 60 ng/ml); DHCR7 (*p* = 0.013 at 0 ng/ml, *p* = 0.006 at 10 ng/ml and *p* = 0.004 at 40 ng/ml); and CYP2R1 (*p* = 0.02 at 0 ng/ml). AMDHD1 was downregulated during simulated orthodontic compressive strain (*p* = 0.006 at 40 ng/ml and *p* = 0.048 at 60 ng/ml). The gene expression data are presented in the Additional file 1: Table S2.

**Fig. 2:**
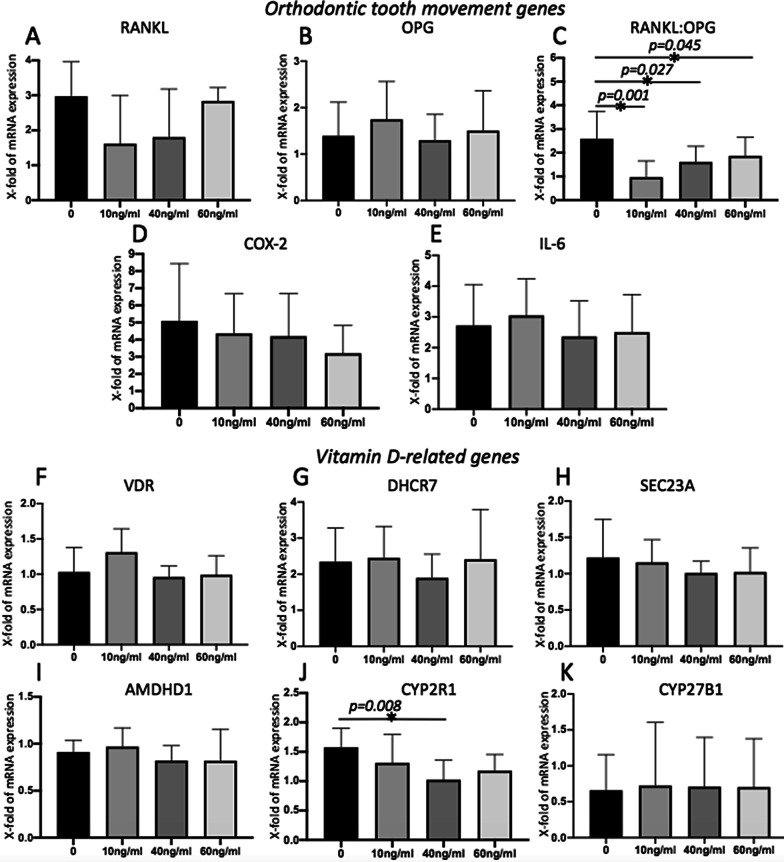
Effects of different concentrations of 25(OH)D_3_ during simulated orthodontic compressive strain on the relative mRNA genes expression levels. **A** Relative RANKL mRNA expression. **B** Relative OPG mRNA expression. **C** Relative RANKL:OPG ratio mRNA expression. **D** Relative COX-2 mRNA expression. **E** Relative IL-6 mRNA expression. **F** Relative VDR mRNA expression. **G** Relative DHCR7 mRNA expression. **H** Relative SEC23A mRNA expression. **I** Relative AMDHD1 mRNA expression. **J** Relative CYP2R1 mRNA expression. **K** Relative CYP27B1 mRNA expression. *On the top of the bars means statistically significant difference (*p* < 0.05)

The Fig. 2 shows the effects of different concentrations of 25(OH)D_3_ during simulated orthodontic compressive strain on the relative expression of RANKL, OPG, the RANKL:OPG ratio, COX-2, IL-6, VDR, DHCR7, SEC23A, AMDHD1, CYP2R1 and CYP27B1. Treatment with 25(OH)D_3_ impacted on the RANKL:OPG ratio levels during simulated orthodontic compressive strain in all evaluated concentrations (10 ng/ml, 40 ng/ml and 60 ng/ml) (*p* < 0.05). 25(OH)D_3_ at a concentration of 40 ng/ml was also associated with CYP2R1 expression (*p* < 0.05).

### The role of different SNPs within the VDR gene for gene expression response (mRNA) to vitamin D

The relative mRNA *VDR* expression according to the genotypes of FokI (rs2228570), BglI (rs739837) and ApaI (rs7975232) in the *VDR* gene and the genotype distribution for each SNP are presented in Table 1. A statistically significant difference was observed in a co-dominant model in the SNP rs7975232. Samples from individuals carrying the CC genotype presented higher relative *VDR* mRNA expression than the samples from individuals carrying the AC genotype under simulated orthodontic compressive strain (pressure).

**Table 1:** VDR mRNA expression [median (minimum–maximum)] according to the genotypes in each studied SNP in VDR

Condition	SNP and experimental concentration	Genotypes median (minimum–maximum)			*p* values	
					Co-dominant model^a^	Recessive model^b^
No pressure	*FokI rs2228570 *(*G* > *A*)	*GG* (n = 12)	*GA* (n = 6)	*AA* (n = 9)		
	0 ng/ml	0.91 (0.01–1.56)	0.98 (0.52–1.64)	1.02 (0.45–1.58)	0.967	0.897
	40 ng/ml	0.85 (0.46–1.77)	0.56 (0.42–0.96)	0.84 (0.28–1.44)	0.173	0.439

	*BglI rs739837 *(*G* > *T*)	*GG* (n = 9)	*GT* (n = 6)	*TT* (n = 12)		
	0 ng/ml	1.01 (0.45–1.58)	1.06 (0.74–1.56)	1.03 (0.52–1.64)	0.852	0.892
	40 ng/ml	0.59 (0.28–0.96)	0.95 (0.81–1.77)	0.85 (0.42–1.44)	0.061	0.038*

	*ApaI rs7975232 *(*A* > *C*)	*AA* (n = 12)	*AC* (n = 9)	*CC* (n = 6)		
	0 ng/ml	1.03 (0.52–1.64)	1.01 (0.75–1.56)	1.09 (0.45–1.58)	0.943	0.922
	40 ng/ml	0.85 (0.42–1.44)	0.84 (0.53–1.77)	0.54 (0.28–0.96)	0.152	0.062

Pressure	*FokI rs2228570 *(*A* > *G*)	*GG* (n = 12)	*GA* (n = 6)	*AA* (n = 9)		
	0 ng/ml	0.82 (0.49–1.72)	1.20 (0.64–3.14)	0.72 (0.47–1.58)	0.248	0.764
	40 ng/ml	0.59 (0.21–0.90)	0.92 (0.60–1.39)	0.84 (0.18–1.15)	0.122	0.235

	*BglI rs739837 *(*G* > *T*)	*GG* (n = 9)	*GT* (n = 6)	*TT* (n = 12)		
	0 ng/ml	0.69 (0.47–2.14)	0.74 (0.49–1.56)	0.86 (0.49–1.72)	0.709	0.451
	40 ng/ml	0.88 (0.18–1.38)	0.68 (0.26–0.90)	0.72 (0.21–1.15)	0.601	0.398

	*ApaI rs7975232 *(*A* > *C*)	*AA* (n = 12)	*AC* (n = 9)	*CC* (n = 6)		
	0 ng/ml	0.86 (0.49–1.72)^a,b^	0.58 (0.47–1.56)^a^	1.29 (0.54–3.14)^b^	0.045*	0.048*
	40 ng/ml	0.73 (0.21–1.15)	0.77 (0.26–0.98)	0.99 (0.19–1.38)	0.683	0.581

*Means statistically significant difference (*p* < 0.05). Comparisons were performed among genotypes within the same SNP under the same experimental conditions. Different letters indicate a significant difference

^a^Kruskal–Wallis with Dunn’s test was used

^b^Mann–Whitney test was used

Statistical significance was also observed in a recessive model. With simulated orthodontic compressive strain (pressure) samples from individuals carrying the CC genotype presented higher relative *VDR* mRNA expression than samples from individuals carrying at least one A allele (AC + AA genotypes) in SNP rs7975232. Without simulated orthodontic compressive strain (no pressure), samples from individuals carrying the GG genotype presented lower *VDR* mRNA expression than samples from individuals carrying at least one T allele (GT + TT genotypes) in rs739837 during treatment with 40 ng/ml of 25(OH)D_3_. With simulated orthodontic compressive strain (pressure) samples from individuals carrying the CC genotype presented higher relative *VDR* mRNA expression than samples from individuals carrying at least one A allele (AC + AA genotypes) in rs7975232.

## Discussion

Although the connection between vitamin D, vitamin D receptor (VDR) and orthodontic phenotypes is not a new subject in the literature and has been investigated by some dental researchers in the past three decades [33], the role of vitamin D as a therapeutical adjunct during OTM is still controversial and the molecular processes occurring during OTM under the influence of vitamin D are largely unknown. Therefore, this in vitro study aimed to investigate, if 25(OH)D_3_ impacts on the expression of some genes involved in the response of hPDL fibroblasts to simulated orthodontic compressive strain. To answer this question, we investigated the effect of different concentrations of 25(OH)D_3_ using an established protocol to simulate OTM in vitro [6]. Additionally, we used cells from different patients, with different genotypes to perform a pilot investigation of the impact of SNPs in *VDR* gene on mRNA expression of OTM-related target genes.

RANKL, OPG, COX-2 and IL6 are genes differentially expressed during OTM [8, 20, 21]. RANKL is well-known as essential for osteoclast formation [37] and binds to the RANK receptor on osteoclast precursor cells initiating osteoclast formation and differentiation [38], while OPG is the endogenous inhibitor of RANKL. Transgenic mice demonstrated that RANKL produced by PDL cells and bone is the major driving force for osteoclastogenesis in response to OTM [36]. Our study confirmed that RANKL was overexpressed during simulated orthodontic compressive strain and also suggested that the RANKL:OPG ratio increases during pressure. The RANKL:OPG ratio regulates osteoclast differentiation, activation and survival and affects the balance between bone formation and resorption [39], which is pivotal for OTM.

The RANKL:OPG ratio is increased during inflammatory conditions. During OTM a biologic response occurs, which is mediated by a variety of inflammatory cytokines described as an aseptic inflammation [3]. In our study both IL-6 and COX-2 expression increased during simulated orthodontic compressive strain. IL-6 has been demonstrated to be overexpressed during OTM [40, 41], which can be attributed to the fact that IL-6 is involved in bone resorption [42], which is required for OTM. Likewise, the expression of COX-2, producing proinflammatory prostaglandins, increases during OTM [6, 40] and is also involved in bone resorption [43].

Many studies highlight important control points in vitamin D molecular pathways. Some of the most remarkable genes include *VDR*, which is the receptor that mediates the action of both 25(OH)D_3_ and 1,25(OH)2D_3_. Other genes, however, are also involved in vitamin D molecular pathways, such as *DHCR7*, SEC23A, *AMDHD1*, *CYP2R1* and *CYP27B1*, which were evaluated here. Interestingly, the expression levels of *DHCR7, AMDHD1* and *SEC23A* changed during simulated orthodontic compressive strain, suggesting that these genes are involved in OTM and the variation in their expression might be involved in clinical outcomes in orthodontic practice. A previous study reported that the conversion of vitamin D to 1,25(OH)2D_3_ in human gingival and hPDL fibroblasts consisted of two steps, in which the conversion from vitamin D3 to 25(OH)D_3_ is under the action of CYP27A1, while the conversion from 25(OH)D_3_ to 1,25(OH)2D_3_ is under the action of CYP27B1 [28]. Although our studied focused on the evaluation of CYP27B1, future studies should also evaluate the role of CYP27A1 during OTM.

The administration of vitamin D during orthodontic treatment has been proposed [33] to accelerate the OTM [10–12] and to enhance tooth position stability [15]. These previous studies are contradicting in their results, as the acceleration of OTM requires a high osteoclast activity, while tooth position stability predominantly requires osteoblastic activity [2]. In our study, different concentrations of 25(OH)D_3_ during simulated orthodontic compressive strain downregulated the RANKL:OPG ratio, which does not support the notion that vitamin D supplementation accelerates OTM. On the contrary, lower levels of the RANKL:OPG ratio should have a decelerating effect on OTM and favor tooth position stability in agreement with Kawakami and Takano-Yamamoto [15].

The role of vitamin D on OTM presented here and in the previous studies [10–12, 15] should be interpreted with caution. Although it is a well-established concept that vitamin D is important for normal development and maintenance of bone and the skeleton, it is also known that supraphysiological doses of vitamin D stimulate bone resorption. The inhibitory effect of vitamin D on RANKL expression occurred only with physiological doses. On the other hand, supraphysiological doses increased Ca^2+^ serum and RANKL expression [44, 45]. Studies observing that vitamin D accelerates OTM performed their experiments injecting high doses of vitamin D [11, 12], whereas in our in vitro study we simulated vitamin D deficiency (10 ng/ml), and vitamin D sufficiency in the lower (40 ng/ml) and higher (60 ng/ml) limits. We did not evaluate supraphysiological levels of vitamin D due the fact that—although it might improve the OTM—we do not want to encourage a therapy with supraphysiological levels with possible vitamin D toxicity.

It is not known, if 25(OH)D_3_ influences the inflammatory response in hPDL cells [14, 30, 31]. In our study, however, COX-2 was not differently expressed among different 25(OH)D_3_ concentrations and IL-6 did not demonstrate to be differentially expressed among them. In previous studies, the production of pro-inflammatory mediators in the PDL was significantly inhibited by vitamin D in a periodontal disease model, including IL-6 [14, 31]. Interestingly, Andrukhov et al. [14] found that 25(OH)D_3_ inhibits IL-6 expression in commercially available hPDL fibroblasts, but not in primary hPDL fibroblasts. Tang et al. [30], Gao et al. [46] and Zhang et al. [47] also observed that vitamin D inhibits IL-8 expression in a periodontal model, but had no effect on IL-6 expression [30]. On the other hand, Nastri et al. [48] observed that IL-6 secretion increased in vitamin-D-treated gingival fibroblasts and hPDL cells exposed to *Porphyromonas gingivalis* and Streptococcus pyogene, while IL-8 secretion decreased.

The action of 25(OH)D_3_ is mediated by its receptor VDR and therefore produces its pleiotropic effects via binding with its ligand and operating a cascade of signaling pathways inducing the activation of various genes [49]. A concentration-dependent correlation was observed between serum vitamin D levels and VDR concentration in the hPDL tissue [32]. Additionally, Andrukhov et al. [14] performed an experiment silencing the *VDR* by siRNA, which resulted in the abolishment of the vitamin D effects in hPDL fibroblasts. The authors concluded that the regulation of expression levels of VDR in hPDL fibroblasts is an important factor influencing functional properties [14]. Although our results do not support that simulated orthodontic compressive strain or 25(OH)D_3_ supplementation influences *VDR* mRNA expression, we decided to perform further analyses in order to evaluate, if SNPs in the *VDR* gene are involved in mRNA expression of this gene in hPDL fibroblasts.

The *VDR* gene is known to exhibit many SNPs [23], which might influence the expression/functions of VDR in hPDL fibroblasts. It is also associated with complex conditions such as OTM. We selected three common SNPs in the *VDR* gene, known as FokI, BgII and ApaI, which have been widely explored in the literature and are associated with different phenotypes/conditions including periodontal disease [25, 26] and external apical root resorption during orthodontic treatment [27]. Our results from individuals carrying different genotypes suggested that both intronic SNPs evaluated here (BgII and ApaI) could be involved in the regulation of *VDR* mRNA expression in hPDL fibroblasts. However, it is important to emphasize that this result is restricted to mRNA data and the protein levels were not assessed. Statistical differences in *VDR* mRNA expression were not observed among the genotypes in FokI—however, this is a missense variation, in which the SNP is responsible for a substitution at exon 2 of the *VDR* gene. As a result of this substitution, the methionine amino acid is translated to threonine, which could finally affect function and efficacy of the VDR protein [50], instead of the expression level. The influence of FokI on transcriptional activation by VDRs in human gingival fibroblasts and hPDL cells has already been investigated in a previous study. After stimulation with vitamin D, the authors observed that the expression of alkaline phosphatase and osteocalcin were different according to the genotypes in FokI [51]. The presence of the wild allele in FokI was also associated with RANKL measurement in patient’s plasma [52]. Therefore, the possibility that SNPs in the *VDR* gene influence gene and protein expression in hPDL cells contributing to the individual difference in OTM velocity cannot be excluded and requires further investigations.

A previous study observed that elevated vitamin D levels were associated with aggressive periodontitis and that these levels were systemically and locally reduced by initial periodontal therapy [53]. Later, this same research group demonstrated that gingival fibroblasts and hPDL cells have 25-hydroxylase activity and convert vitamin D to 1,25(OH)2D_3_ [28]. Therefore, it is possible that OTM has a possible local and/or systemic effect on vitamin D levels and this topic should also be investigated in future clinical studies.

Briefly, our results do not support that physiological concentrations of 25(OH)D_3_ may enhance the OTM, however it is possibly involved in the reestablishment of bony tissue supporting the teeth after OTM. Also, our results do not support that vitamin D deficiency leads to a slower rate of OTM, as proposed in a recent review [33].

## Conclusion

Simulated orthodontic compressive strain impacted on the expression of OTM- and vitamin-D-related genes. Physiological concentrations of 25(OH)D_3_ are involved in gene expression variations during simulated orthodontic compressive strain. SNPs in *VDR* gene may affect mRNA expression of VDR in hPDL fibroblasts.

## Supplementary Information

**Additional file 1.** Studied target genes and reference genes.

## Data Availability

All data generated or analyzed during this study are available from corresponding authors to any reader directly upon reasonable request.

## Abbreviations

PDL: Periodontal ligament
hPDL: Human periodontal ligament
OTM: Orthodontic tooth movement
PCR: Polymerase chain reaction
RANKL: Receptor activator of nuclear factor-κB ligand
OPG: Osteoprotegerin
COX-2: Cyclooxygenase-2
IL-6: Interleukin-6
VDR: Vitamin D receptor
DHCR7: 7-Dehydrocholesterol reductase
SEC23A: Sec23 homolog A
AMDHD1: Amidohydrolase domain containing 1
CYP27B1: Cytochrom P450 familie 27 subfamilie B polypeptid 1
CYP2R1: Cytochrome P450 family 2 subfamily R member 1
RPL22: Ribossomal protein L22
PPIB: Peptidylprolyl isomerase B
CYP27A1: Cytochrome P450 family 27 subfamily A member 1
